# Machine Learning-Enhanced Evaluation of Handheld Laser-Induced Breakdown Spectroscopy (LIBS) Analytical Performance for Multi-Element Analysis of Rock Samples

**DOI:** 10.3390/s26031076

**Published:** 2026-02-06

**Authors:** Giorgio S. Senesi, Olga De Pascale, Ignazio Allegretta, Roberto Terzano, Bruno Marangoni

**Affiliations:** 1 CNR-Istituto per la Scienza e Tecnologia dei Plasmi (ISTP) Sede di Bari, Via Amendola, 122/D, 70126 Bari, Italy; olga.depascale@istp.cnr.it; 2 Dipartimento di Scienze e Tecnologie Biologiche ed Ambientali, Università del Salento, Via Monterioni 165, 73100 Lecce, Italy; ignazio.allegretta@unisalento.it; 3 Dipartimento di Scienze del Suolo, della Pianta e degli Alimenti, Università degli Studi di Bari, Via G. Amendola 165/A, 70126 Bari, Italy; roberto.terzano@uniba.it; 4 Programa de Pós-Graduação em Ciência dos Materiais, UFMS–Universidade Federal de Mato Grosso do Sul, Av. Costa e Silva, s/nº, Campo Grande 79070-900, Brazil; bruno.marangoni@ufms.br

**Keywords:** handheld LIBS instruments, certified geochemical reference materials, PLS, RF, ANN

## Abstract

Handheld laser-induced breakdown spectroscopy (hLIBS) can be considered one of the most recent techniques for rock characterization in situ. Handheld LIBS devices are useful tools for providing “fit for purpose” qualitative and quantitative geochemical data. The analytical performance of hLIBS instruments varies significantly between similar instruments from different manufacturers. This study employed two commercial hLIBS instruments, both making use of noise reduction and multivariate partial-least-squares (PLS) calibration. Model validation was performed using the Leave-One-Out Cross-Validation (LOOCV) method. The Random Forest (RF) and Artificial Neural Network (ANN) algorithms were also employed as complementary approaches to PLS modeling, with the goal of exploring potential nonlinear relationships between spectral intensities and reference analyte concentrations. A comparison was also made with the most basic and commonly used approach, univariate analysis, demonstrating that multivariate methods achieve superior performances. To evaluate the predictive performance and quantification capability of the acquired LIBS spectra, the Pearson’s coefficient (R^2^) and root-mean-square error (RMSE) were employed in the analysis of 21 diverse certified geochemical reference materials (CRMs). The results achieved suggested that the spectral resolution was the key factor determining the performance of multivariate LIBS calibrations. The PLS model proved to be satisfactory for analyses performed by the higher-spectral-resolution instrument, whereas complementary algorithms were necessary to achieve better results with the lower-spectral-resolution instrument.

## 1. Introduction

In geoscience research, the need for chemical data has continuously expanded with regard to not only the type of elements and their concentration levels but also to the extension of applications to a wider and wider variety of geological materials. A persistent requirement by the geoscience community is the availability of field-deployable instrumentation that can be used regularly outside the laboratory to perform in situ chemical analysis. The basic requisites of these instruments include ease portability, lightweightness to enable individual operation, durability and robustness for use in challenging environments, user friendliness, and the ability to detect the broadest spectrum of elements in a brief acquisition time [1,2].

Laser-induced breakdown spectroscopy (LIBS) and X-ray fluorescence spectrometry (XRF) are two analytical methods that meet the above-listed requisites for field use under current environmental conditions, enabling exploration geologists to acquire an immediate insight into the geochemistry of an ore prospect. In this way, field survey work can be optimized, and field-acquired data can assist in deciding which samples to gather for additional laboratory examination, thereby offering significant savings in both time and expense. Furthermore, data acquired in the field allow environmental geologists to quickly understand the existence of pollution by hazardous elements throughout an area and/or assess the effectiveness of a remediation effort to restore a contaminated site [1], identify the possible presence of precious or useful elements [2], and explore mine wastes on site in view of their possible recovery [3].

LIBS is one of the very few current analytical technologies suitable for routine use outside the laboratory and possesses a persuasive set of advantages that makes it ideally suited for chemical analysis in the field [2]. These include the possibility of using a compact and lightweight instrument operated by a single individual to analyze most types of natural materials under ambient environmental conditions in real time, and with little to no sample preparation [2]. Although portable LIBS instruments may not achieve the same levels of elemental detection and analytical accuracy as those of laboratory instruments, they still offer an effective and robust tool for the field investigator. Currently, LIBS is being extensively utilized in various geoscience sub-disciplines, including mineralogy, petrology, volcanology, sedimentology, the exploration and exploitation of natural resources, pedology, and geoarchaeology [4,5,6].

In the first decade of the current millennium, the progress in the analytical performances of small and compact LIBS analyzers has been significantly influenced by the choice of LIBS as one of the technologies incorporated into the NASA ChemCam system on the Curiosity rover to assess the elemental composition of Martian soils and rocks [7]. Subsequently, in 2013, the initial handheld (h) LIBS analyzer was launched in the commercial sector and was reported in the scientific literature soon after [8]. While the progress of portable LIBS equipment for geological use has advanced in research laboratories e.g., [9,10,11], commercial hLIBS analyzers have become the favored response to the ongoing demand for technology that enables quick chemical analysis in field settings.

The aim of this study was to analyze the reliability and precision of two commercial hLIBS instruments operating in different configurations (i.e., varying pulse energies and durations, acquisition geometries, spot sizes, raster grids, repetition rates, and spectral ranges) in analyzing 21 diverse certified geochemical reference materials (CRMs). Besides evaluating the general applicability of hLIBS for the geochemical analysis of rocks, the effect of multiple rock characteristics on the hLIBS performance was also investigated. In order to verify the performances of the two instruments, noise reduction and multivariate partial-least-squares (PLS) calibration and validation Leave-One-Out Cross-Validation (LOOCV) were used. For each test sample in the LOOCV, hyperparameter optimization was conducted using nested cross-validation (NCV) to avoid overfitting [12,13]. The Random Forest (RF) and Artificial Neural Network (ANN) algorithms were also employed as complementary approaches to PLS modeling, with the goal of exploring potential nonlinear relationships between spectral intensities and reference analyte concentrations [14,15,16]. Univariate analysis was employed for quantitative purposes as a benchmark against the multivariate approaches, highlighting the performance achieved. The final aim of this work is to assess the robustness of hLIBS in general, and the intention is not to suggest the best instrument but rather the range of applicability of two types of common commercial instruments.

## 2. Materials and Methods

### 2.1. Certified Geochemical Reference Materials

A total of 21 homogenized rock samples originating from 10 different countries were examined in this study. The raw materials to obtain these CRMs originated from various deposits spread across the globe (Service d’Analyse des Roches et des Minéraux-SARM). The goal was to offer the scientific community CRMs adapted to geochemical analysis and necessary for the calibration of a device or the evaluation of a measurement method. An overview of the samples, along with their specific rock characteristics and elemental concentrations, can be found in the Supplementary Materials (Tables S1 and S2). The CRM samples were air-dried and ground to achieve particle sizes < 20 µm. The pellets to be used for the analysis were obtained by mixing 5.0 g of powdered sample with 2 mL of 2% (*w*/*v*) Elvacite^®^ 2046 (PANalytical B.V., Almelo, The Netherlands) acetone solution in a mortar, stirring the slurry until the acetone evaporated, and finally pelletizing the powder placed in 30 mm open-ended aluminum cups by uniaxial pressing (25 tons).

### 2.2. Handheld LIBS Instruments Used and LIBS Spectrum Collection

In this work, a NanoLIBS hLIBS instrument (B&W Tek, Newark, DE, USA) described in detail by Senesi et al. [17] was used. The entire apparatus has dimensions of 26 × 10 × 30 cm, a weight of 1.8 kg, is powered by an onboard rechargeable Li-ion battery (8 h battery life) and operates at ambient atmosphere. The instrument employs a 1064 nm Nd:YAG miniature-diode-pumped solid-state short-pulsed laser that produces a focused beam of 20–30 μm that delivers a maximum pulse energy of 150 μJ to the sample at a pulse duration of 500 ps for a shot repetition frequency between 1 and 5 KHz.

The NanoLIBS hLIBS instrument allows for the scanning of a spectral range from 180 to 800 nm with an overall resolution of 0.4 nm (lower-spectral-resolution instrument). The emission light signal is collected and transferred via a fiber optic cable into a compact low-resolution spectrometer operating in non-gated mode. The system is equipped with a raster scan that provides spatial averaging. Measurements were performed by placing the instrument against the surface of the pellet, and the analysis was initiated via a trigger. Spectral data were collected at an integration time of 4 msec, which corresponded to twelve laser pulses for a 1 kHz laser. Five different positions on each pellet were analyzed, and five laser measurements were acquired at each position, which were then averaged to obtain a single spectrum for each position. The spectra were then analyzed qualitatively to identify and highlight the emission lines using the National Institute of Standards and Technology (NIST) Atomic Spectra Database [18].

The other instrument employed in this study was a Z-300 (SciAps, Inc., Woburn, MA, USA) LIBS device, measuring 21 × 29 × 11 cm, weighing 1.8 kg, and operating by an internal Li-ion battery. This device utilizes a Class 3 PULSARTM 1064 nm Nd:YAG diode-pumped solid-state pulsed laser, generating a 100 µm focused beam that fires a 5–6 mJ pulse to the sample with a 1 ns pulse duration at a repetition rate of 1 to 50 Hz. The analyzer operates in ambient air but can also perform gas purging by supplying an inert gas (Ar) from a canister in the instrument handle directly to the focusing area on the sample surface where LIBS plasma is formed, thereby enhancing plasma containment and emission signals.

The Z-300 instrument captures a wide spectrum of plasma light emissions, spanning from 190 to 950 nm. The emission light signal is gathered and transmitted through a fiber optic cable to three internal spectrometers that use time-gated CCD detectors, which have spectral ranges and resolutions of 190–365 nm with a full width at half maximum (FWHM) of 0.18 nm, 365–620 nm with a FWHM of 0.24 nm, and 620–950 nm with a FWHM of 0.35 nm (higher-spectral-resolution instrument). Spectral data were gathered with a delay time of 650 ns over an integration period of 1 ms. The routine wavelength calibration of the spectrometers was conducted by examining an internal target composed of Grade 316 Mo-bearing stainless steel. For each spectrometer in the system, discrepancies in wavelengths between the chosen observed emission lines and the data obtained from the NIST database [18] were identified, and correction factors were utilized. The updated coefficients remained in use until the subsequent wavelength calibration occurred, and a stored record of all calibration spectra and adjustment values was maintained onboard. The analyzer is equipped with a video camera to observe the sample prior to analysis and a laser spot finder that indicates where the laser beam is hitting the sample. The instrument features a computer-controlled, onboard 3D translational stage that automates laser focusing on the sample site and allows for rastering the laser beam across the sample in the XY-direction at specific positions for targeted analysis or data averaging. The raster pattern was obtained at intervals of 12.5 µm across an area of up to 2 × 2 mm^2^, with both the grid size and number of laser shots at each raster point fixed by the user. Furthermore, it was possible to use a number of non-analytical shots for surface “cleaning” before gathering the spectral data.

Obviously, the two hLIBS instruments feature different configurations that are reflected in the resulting spectra. The comparison in Table 1 reports an overview of the main characteristics of the two hLIBS instruments. LIBS analysis was undertaken averaging four spectra recorded after one cleaning shot at a laser-firing rate of 50 Hz over a 4 × 3 grid for each pellet. This array sampling was repeated five times on random positions on each pellet.

### 2.3. Pre-Analyses and Limit of Detection (LOD)

The raw spectra initially underwent a preprocessing pipeline. This sequence consisted in: (i) background removal using a polynomial fitting method; (ii) outlier detection via the Spectral Angle Mapper (SAM) algorithm; and (iii) normalization by Standard Normal Variate (SNV) [19]. Finally, the five-position spectra acquired for each pellet were averaged. In this specific study, the SAM analysis did not exclude any spectra as outliers, and thus all acquired data were retained for subsequent analysis.

An additional preprocessing step was applied prior to feeding the data to the machine learning routines. To reduce the number of initial variables (wavelengths) and improve the training efficiency, an automatic correlation-based variable selection procedure was implemented to retain the most informative spectral features. First, the Pearson’s coefficient (R^2^) between the intensity of each wavelength and the reference concentration of the constituent was calculated to exclude from the dataset the wavelengths exhibiting R^2^ values lower than 0.3 [20]. This threshold was chosen based on the empirical observation that most of the uncorrelated transitions and background noise lay below this value. Subsequently, a second filtering routine was used to compare the selected spectral variables with a list of known atomic and ionic transitions for the element or oxide of interest. Wavelengths that were not located within a ±1 nm window of a registered emission line for the corresponding element were removed from the analysis. The electronic transitions for each element were extracted from the NIST LIBS database [18], and the strongest transitions were selected, with a maximum of 30 transitions (atomic and ionic), considering a plasma condition at a temperature of 1 eV and an electron density of 10^17^ cm^−3^.

The limit of detection (LOD) was determined using a univariate calibration curve that related the intensity variation of a specific spectral transition to the reference concentration of the analyte in the sample. A linear model was adjusted, and the slope of the regression line was obtained. The standard deviation of the background noise (std) was estimated from a clean spectral region with no corresponding emission line [21]. According to the criteria established by the International Union of Pure and Applied Chemistry (IUPAC), the LOD was calculated as:

LOD = 3std/slope(1)

The LOD values obtained in this study were consistent with those reported in the literature for LIBS systems applied to geochemical standards with vast compositional variety that include unique natural (igneous, sedimentary, and metamorphic) samples [22]. Table 2 and Table 3 present the figures of merit, R^2^, standard deviation of the background (Std background) and slope of the regression line used for the calculation of the LOD. The corresponding univariate calibration and validation curves are provided in the Supplementary Materials (Figures S1–S8). After this stage, samples with concentrations below the LOD were excluded in order to ensure the statistical robustness of the models (PLS, RF and ANN). This criterion ensures that the model maintains adequate complexity to capture relevant relationships while minimizing the likelihood of overfitting to the calibration data.

### 2.4. Multivariate Analysis

To assess the calibration and quantification performances of the two LIBS systems, three distinct analytical approaches were employed: PLS, ANN, and RF. The choice of these methods was based on their complementary characteristics. In particular, PLS is widely used in spectroscopy because it efficiently handles highly correlated and noisy data while exploiting the existing linear relationships between spectral intensities and analyte concentration variations [23]. This approach is particularly advantageous when the number of spectral variables exceeds the number of samples, as it enables dimensionality reduction without the significant loss of relevant chemical information.

Differently, the machine learning-based methods RF and ANN exhibit a superior capacity to model nonlinear relationships between spectral variables and concentrations. In particular, the RF algorithm is composed of an ensemble of decision trees; thus, it is robust to noise and less prone to overfitting. Furthermore, it provides metrics of variable importance that facilitate the interpretation and identification of the most relevant emission lines. In turn, ANNs offer high flexibility in capturing complex patterns and variable interactions, making them suitable for scenarios in which the spectral response exhibits nonlinear behavior as a function of the analyte concentration [24]. The combined use of these approaches allows for a comprehensive analysis, capable of capturing both linear and nonlinear trends in the data.

Univariate validation analysis was also performed using the same criteria established for the multivariate analysis. This approach was adopted as a benchmark against multivariate methods, enabling a direct comparison and allowing the effectiveness of applying multivariate analysis to this set of samples and experimental setup to be properly assessed.

To evaluate the predictive performance and quantification capability of the acquired spectra, the R^2^ and root-mean-square error (RMSE) were employed. Model validation was performed using the LOOCV method, from which the RMSE and R^2^ values were extracted.

To ensure a robust and unbiased evaluation of the model performance while mitigating the risk of overfitting, a nested cross-validation (NCV) procedure within the LOOCV procedure was implemented and employed to perform the selection of hyperparameters for each model’s training [25]. This approach is particularly advantageous for smaller datasets, as it provides a reliable estimate of a model’s predictive capability on unseen data [26]. The methodology consists of two hierarchical loops: an external loop and an internal loop. The external loop employs LOOCV, where each individual sample in the dataset is sequentially held out as an independent test set. For each iteration of this external loop, the remaining N-1 samples form the training set. Within this training set, an inner 5- or 10-fold cross-validation loop is executed to perform the hyperparameter optimization. This internal loop conducts a grid search over a pre-defined parameter space to identify the optimal configuration that maximizes the model performance on the internal validation folds, ensuring that the model is adjusted without any information from the held-out test sample leaking into the training process. Once the optimal hyperparameters are identified, a final model is trained on the entire N-1 training set using these parameters, and then the model is used to predict the held-out test sample. The cycle described above is repeated until every sample has served as the test sample once [27]. The most frequently selected parameters are then chosen and used to perform a LOOCV procedure. The predictions from all LOOCV iterations are subsequently aggregated and compared against the reference values. A table detailing the optimal hyperparameter values selected for each classifier (PLS, RF, and ANN) during the LOOCV procedure is provided in the Supplementary Materials Table S3.

The overall performance of the modeling strategy was quantitatively assessed by constructing a validation plot of predicted versus real concentrations and by calculating the R^2^ and RMSE. Although computationally demanding, this procedure is feasible with our sample size and effectively prevents overfitting by strictly separating the data used for model tuning from those used for performance assessment. In this work, the specific hyperparameters optimized using NCV were the number of latent variables for the PLS; the number of trees and number of leaf nodes for the RF; and, for the ANN, the number of neurons, number of hidden layers, and number of PCA latent variables used as input features. The ANN was performed with 100 epochs with a fixed learning rate of 0.01 (e.g., Figures S9 and S10 in the Supplementary Materials, where the RMSE for the calibration and validation curves for the CaO and MgO analyses in the lower-spectral-resolution instrument are shown).

The LOOCV approach consists of a cross-validation process in which, at each iteration, a single sample is excluded from the calibration set and used as a test sample. This procedure is repeated successively until every sample is used once for testing. The main advantage of LOOCV lies in its maximal utilization of available data, which is particularly beneficial for datasets with limited sample sizes, thereby providing a robust estimate of the model’s generalization capability.

The RMSE represents the square root of the mean of the squared differences between the predicted values generated by the model and the experimental reference values. This parameter quantifies the predictive accuracy of the model, i.e., smaller RMSE values indicate a higher predictive capability and lower error dispersion. The coefficient of determination (R^2^) complements this metric by quantifying the proportion of the variance in the reference data that is explained by the model. Higher R^2^ values denote better agreement between predicted and measured values, reflecting the model’s ability to capture the underlying data structure. However, the R^2^ alone may overestimate the model performance, particularly in cases of model overfitting or when the variance of the target variable is high. While the RMSE directly measures the predictive error magnitude, the R^2^ evaluates the explanatory power of the model. Thus, used together, the RMSE and R^2^ provide a more comprehensive and reliable assessment of the model performance. Their combined use ensures a balanced interpretation between goodness of fit and predictive performance, reducing the likelihood of overestimating the model quality due to overfitting effects. All routines used in this work were developed by the authors using Matlab2016b.

### 2.5. Partial-Least-Squares (PLS) Modeling

The PLS regression approach was employed to correlate the spectral intensities with the reference concentrations of the analytes. PLS is a multivariate statistical method widely used in spectroscopy, as it combines dimensionality reduction and predictive modeling within a single framework [20]. The algorithm projects both the spectral variables (matrix X) and concentration variables (matrix Y) into a new space of latent variables to maximize the covariance between X and Y. These latent variables thus retain the most relevant information for prediction while minimizing noise and multicollinearity, which is a challenge in LIBS spectral datasets.

The final dataset, comprising 21 samples at maximum, was used for the initial model training. Samples with excessively high concentrations were excluded to avoid biasing the calibration and the dominance of extreme points (see the Supplementary Materials (Table S2)). The optimal number of latent variables was determined by maximizing the R^2^ calibration in the NCV phase. The model performance was optimized using NCV and validated by the LOOCV procedure. As figures of merit, the R^2^ and RMSE for LOOCV validation were computed to quantitatively assess the model performance.

### 2.6. Random Forest and Artificial Neural Network Modeling

The RF and ANN algorithms were employed as complementary approaches to PLS modeling, with the goal of exploring potential nonlinear relationships between spectral intensities and reference analyte concentrations. Both algorithms were implemented following a prior spectral selection of the most intense transitions, based on the NIST LIBS database, which provided reliable information on the strongest emission lines of each element [18].

The RF algorithm is an ensemble learning method that constructs multiple independent decision trees from random subsets of the calibration data and spectral variables. Each tree performs an individual prediction, and the final output is obtained as the average (in regression tasks) of all tree predictions. This ensemble approach reduces the risk of overfitting and enhances model robustness against experimental noise. The parameter optimization for the RF model was conducted by varying both the number of trees (estimators) and maximum leaf nodes.

ANNs are models inspired by the structure and functioning of the human brain; i.e., they are composed of multiple layers of interconnected artificial neurons. Each neuron applies a nonlinear activation function to its input data, allowing the model to capture complex and nonlinear dependencies between spectral variables and analyte concentrations [28].

For the ANN analyses, a Principal Component Analysis (PCA) preprocessing step was performed. PCA is a statistical technique that transforms the original set of correlated variables into a smaller number of uncorrelated components, known as principal components. This transformation preserves most of the variance in the dataset while reducing its dimensionality. The use of PCA is particularly important in spectroscopic and multivariate analysis because it removes redundant information, mitigates collinearity issues, and highlights the most prominent variance patterns. By reducing the number of variables while retaining the essential spectral features, PCA contributes to faster model training and can improve the overall stability and generalization ability of machine learning models. This step was essential for performing the ANN training, as using the raw wavelength intensities directly would have made the routine computationally prohibitive for the analysis.

**Figure 1:**
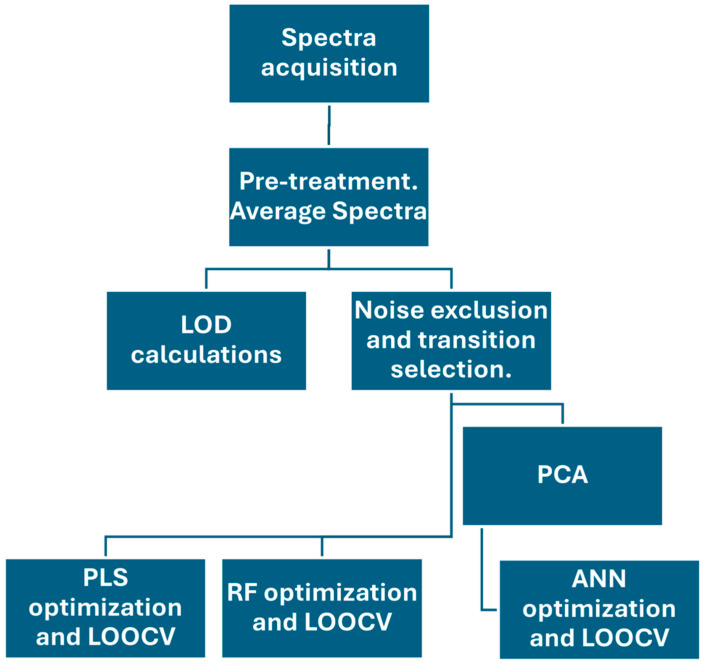
Schematic overview of data treatment and analysis sequence employed.

The hyperparameter optimization and validation process applied to both the RF and ANN models was the same of that employed for the PLS, i.e., NCV and LOOCV. As figures of merit, the R^2^ and RMSE obtained were calculated for the validation curves. The model parameters, which include the numbers of trees, leaves, neurons and hidden layers, were systematically adjusted to maximize the calibration R^2^ while monitoring the RMSE in the NCV phase, thereby ensuring the stability, reproducibility, and statistical consistency of the models. Samples with concentrations below the LOD were excluded in order to ensure the statistical robustness of the model. A flowchart illustrating the data processing and analysis pipeline is presented in Figure 1.

## 3. Results and Discussion

### 3.1. Results Achieved by the Higher-Spectral-Resolution Instrument

**Figure 2:**
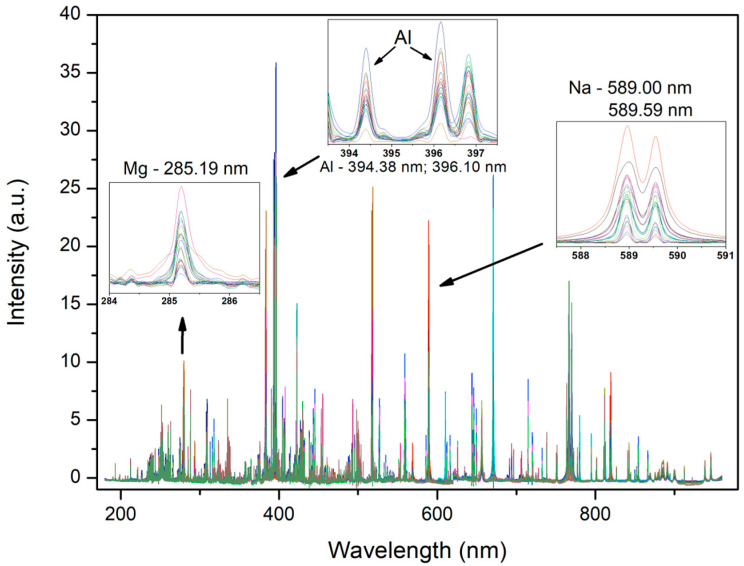
Averaged LIBS spectra acquired for all samples with the higher-spectral-resolution instrument.

The processed spectra acquired by the higher-spectral-resolution instrument are shown in Figure 2. This figure provides a comparative visualization of selected atomic emission lines across different samples. The electronic transitions of Mg, Al, and Na are highlighted, clearly illustrating the variation in the signal intensities corresponding to the concentration differences among the samples.

The results summarized in Table 4 show that the higher-spectral-resolution hLIBS system exhibits a good analytical performance and was able to quantify 16 out of the 25 evaluated analytes in the samples, including most of the major oxides and several trace elements. Nine analytes presented non-trustable results (MnO, P_2_O_5_, CO_2_, SO_3_, F, Cl, Ni, Cu, Zn), which are excluded from Table 4, and could not be reliably characterized because their concentrations were below the LOD or their emission lines exhibited significant spectral interference that prevented a reliable calibration.

Data in Table 4 also show that the achieved LOD values fall within the expected range for samples of similar composition [22], which confirms that the instrumentation employed is suitable for quantitative analyses of complex materials, such as rocks of different origins and compositions. Furthermore, high R^2^ values and low RMSEs were achieved for most of the major oxides, with good performances for Al_2_O_3_, MgO, CaO, Na_2_O, and K_2_O, which exhibited a strong linear correlation between their spectral intensities and reference concentrations.

The three algorithms demonstrated distinct but complementary performances. In particular, the PLS algorithm provided a consistent performance across most analytes, thereby confirming its effectiveness in modeling predominantly linear relationships. Differently, the RF and ANN appeared to be more effective at capturing nonlinear dependencies, particularly for minor elements and analytes affected by multiple spectral interferences. This complementarity highlights the advantage of combined (multi-algorithm) modeling approaches in achieving more robust and generalizable LIBS calibrations.

In conclusion, the higher-spectral-resolution LIBS system demonstrated a broad and precise analytical capability, allowing for the identification of both major oxides and trace elements with high reliability.

Overall, the PLS model exhibited the best performance in the quantification of the investigated elements and oxides. For some analytes, i.e., CaO, TiO_2_, Ba and Sr, PLS provided inferior but comparable results, although its predictive accuracy was nearly equivalent. Only for Ba and Sr did the ANN model demonstrate a clearly superior performance. This discrepancy might be attributed to electronic transition interferences or co-dependencies arising from fluctuations of other constituents, a phenomenon also reported in similar studies [22]. Moreover, the strategies adopted here for hyperparameter optimization and validation substantially minimize the risk of overfitting [26], thereby ensuring a more reliable estimation of the model’s predictive capability. For the analysis of TiO_2_, the results appear limited, indicating the need for more samples with a broader concentration range for further investigation.

**Figure 3:**
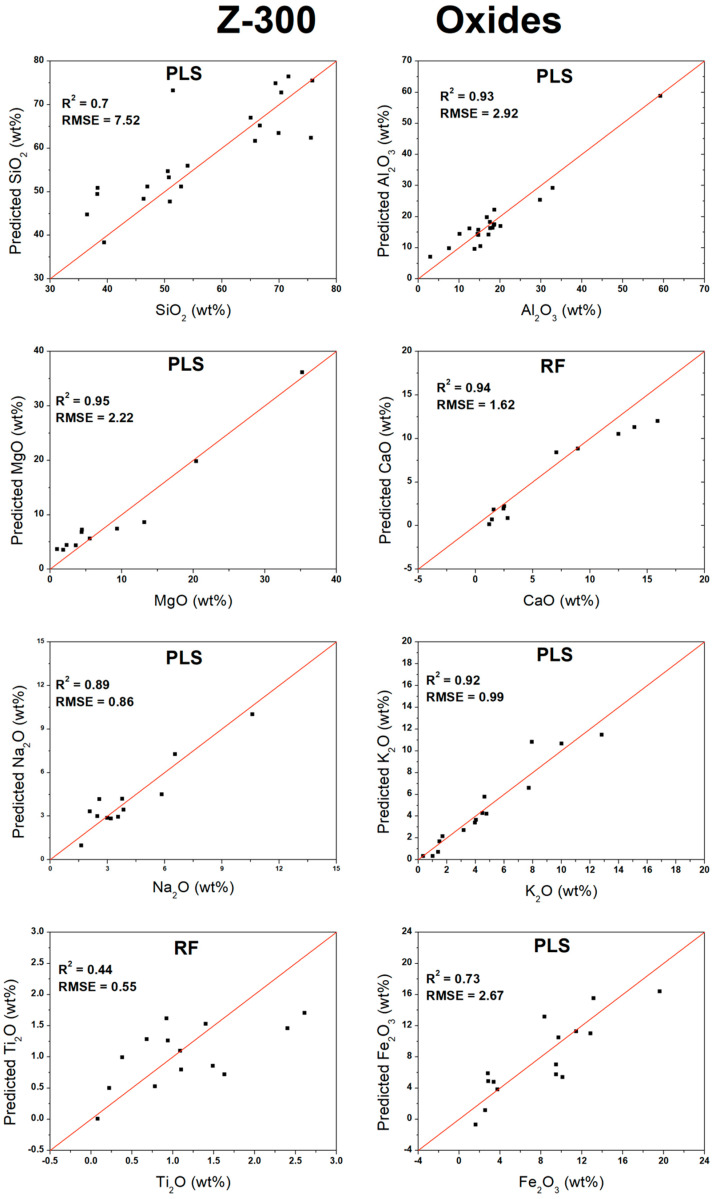
Validation curves for oxides measured by higher-spectral-resolution instrument.

**Figure 4:**
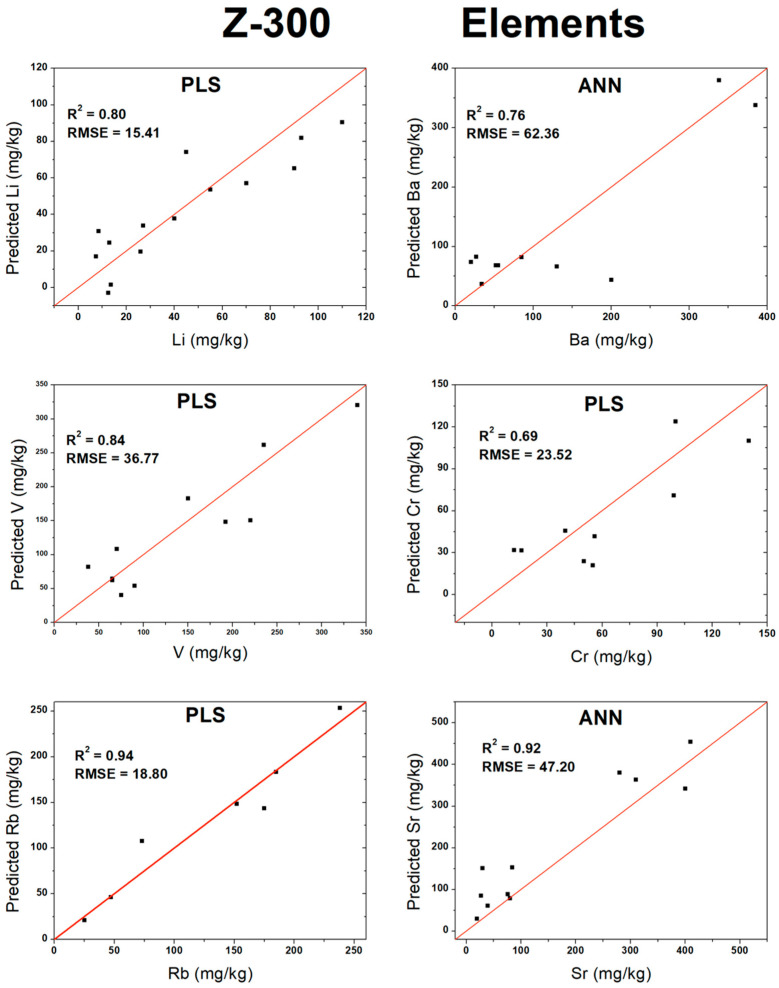
Validation curves for atomic elements measured by higher-spectral-resolution instrument.

Based on the optimal results achieved for each algorithm (PLS, RF, or ANN) from the validation in Table 4, scatter plots were constructed comparing the predicted analyte concentration against the actual measured concentration. For most measurements, the multivariate analyses yielded superior results when compared to the univariate approach. The only exception was K_2_O, for which a slight improvement was observed using univariate analysis, with the R^2^ increasing from 0.92 to 0.96 and the RMSE decreasing from 0.99 to 0.71 wt%. In this specific case, the selected emission line (693.63 nm; Table 2) effectively captured the concentration-dependent behavior. Additionally, the oxides MgO, CaO, and Na_2_O, as well as the element Rb, also produced satisfactory results under univariate analysis, although their performances remained slightly inferior to those achieved with the multivariate methods. Figure 3 presents the validation plots for the oxides, while Figure 4 displays the corresponding plots for the elements.

### 3.2. Results Achieved by the Lower-Spectral-Resolution Instrument

**Figure 5:**
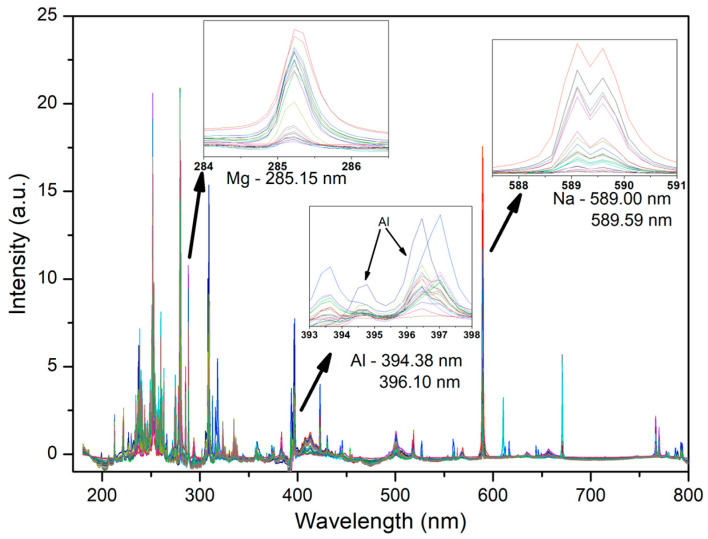
Averaged LIBS spectra for all samples acquired with lower-spectral-resolution instrument.

Figure 5 presents the acquired averaged LIBS spectra for the lower-spectral-resolution instrument, highlighting the electronic transitions of Mg, Al, and Na. The intensity variations of these emission lines correspond to concentration differences among the samples.

The lower-spectral-resolution instrument was able to satisfactorily quantify only 11 out of the 25 analytes (Table 5), using the same calibration and validation criteria. Nevertheless, the lower-spectral-resolution instrument still provided very good results for Al_2_O_3_, MgO, CaO, and Na_2_O, with R^2^ values above 0.9, which indicates that, despite its optical limitations, the system can still be useful for process control applications or the rapid screening of homogeneous samples. In particular, the analyte K_2_O showed a poorer performance for all models, likely because its main emission line (≈766 nm) lies near the upper end of the instrument’s spectral range, where detector gain and sensitivity decrease. This effect is well known and reported in the literature as a common limitation of spectrometers equipped with short-range linear CCD detectors [29]. TiO_2_, Fe_2_O_3_, and Cr show limited results, indicating that further studies are required to improve their performances. Be and Pb, although also limited, exhibited results that indicate potential for future measurements. Overall, the multivariate analyses once again outperformed the univariate approach. The only exception was Cr, for which the R^2^ increased from 0.48 to 0.63 when using univariate analysis. Nevertheless, this improvement remains limited, indicating that the quantitative analysis for low-concentration elements is constrained when using a low-resolution spectrometer. Reasonable results were obtained with univariate analysis for the oxides Al_2_O_3_, MgO, and Na_2_O, suggesting that univariate approaches may still be applicable for these specific cases.

**Figure 6:**
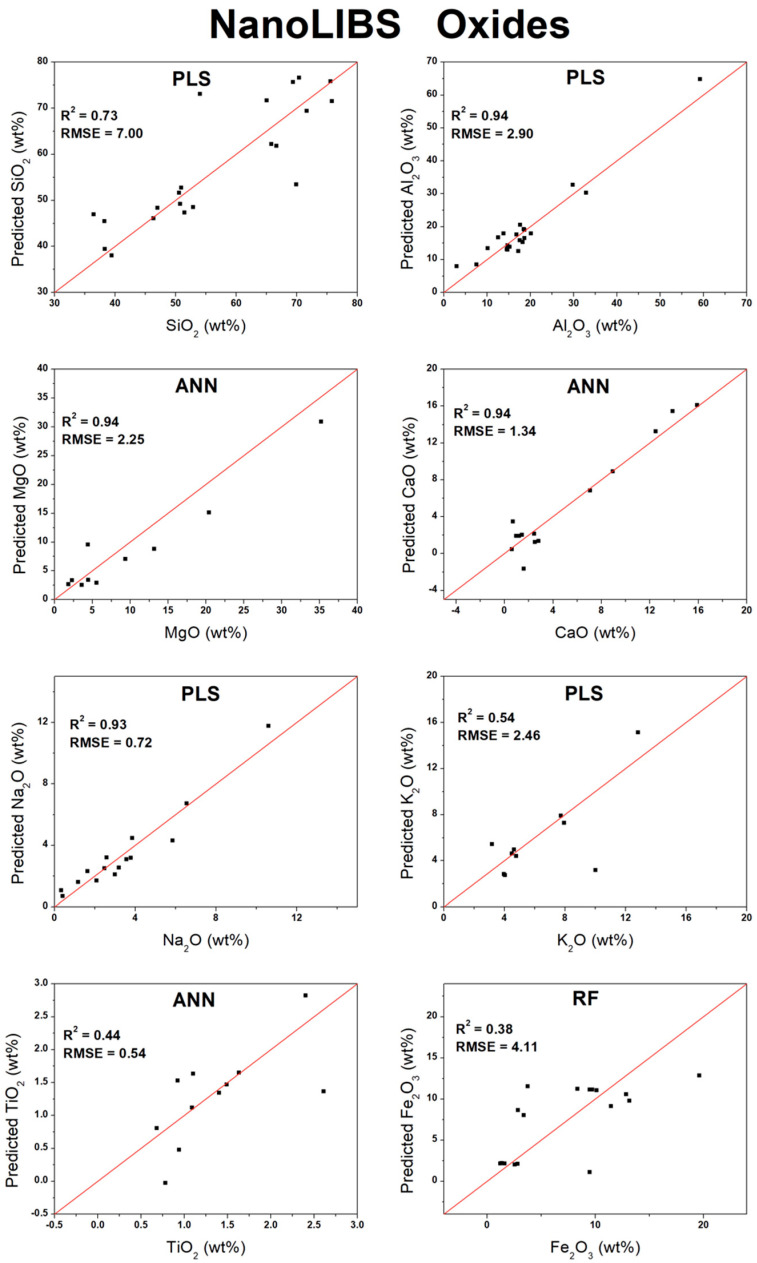
Validation curves for oxides measured by lower-spectral-resolution instrument.

**Figure 7:**
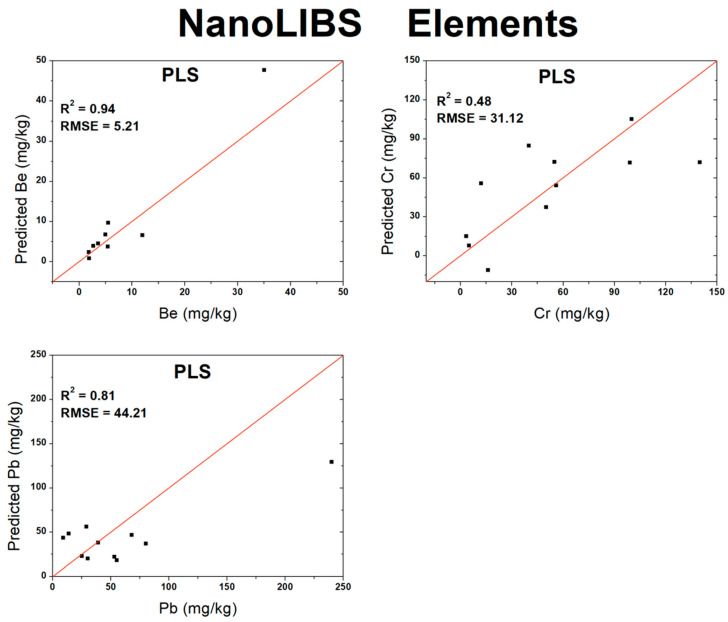
Validation curves for atomic elements measured by lower-spectral-resolution instrument.

Based on the optimal results achieved for each algorithm (PLS, RF, or ANN) from the validation shown in Table 5, scatter plots were constructed comparing the predicted analyte concentrations against the measured concentrations. Figure 6 presents the validation plots for the oxides, while Figure 7 displays the corresponding plots for the elements.

### 3.3. Complementary Performances of the Two Instruments

The results obtained indicated that, as expected, the two instruments feature different analytical performances, which suggests that a higher spectral resolution and optical sensitivity are critical for improving the analytical accuracy and quantification capability. In particular, the spectral resolution was the key factor determining the performance of multivariate LIBS calibrations. A direct comparison of the spectra acquired with the two instruments (Figure 2 and Figure 5) reveals a clear difference in the spectral resolutions between them, highlighting their different analytical capabilities. The main limitations were related to the lower spectral resolution and reduced signal-to-noise (SNR) ratio, which hinder the discrimination of closely spaced spectral lines and increase the overlap of transitions. These limitations directly affect the system’s ability to perform automatic modeling and reduce the reliability of multivariate calibrations, mainly for trace elements. While one instrument was able to model both major oxides and trace elements with good stability, the other system exhibited some limitations in sensitivity and selectivity, especially for low-concentration analytes. However, one point that should be highlighted is the ability of the lower-resolution instrument to measure Pb and Be, with some limitations, which is an outcome that the higher-resolution system was unable to achieve. This difference may be associated with intrinsic factors, such as spectrometer gain settings or the greater ease of data processing under these conditions. It should also be noted that, in this case, fewer samples were analyzed and, specifically for Be and Pb, the concentration distributions were not homogeneous, which may have influenced the results. In this context, the findings indicate the potential for application; however, a larger number of samples must be included to ensure greater robustness.

Another possible cause of the different performances of the two instruments can be attributed to the different laser raster grids used; i.e., while the one has a fixed grid, the other allows the user to modify the grid, thereby improving its number. However, the different spectral ranges of the two instruments appeared to not have affected their overall performances.

In particular, the LODs achieved with the higher-spectral-resolution system were systematically lower for most of the reported elements. For instance, the LOD for K_2_O decreased from 2.21 wt% (lower-resolution system) to 0.35 wt% (higher-resolution system), and for TiO_2_, these values decreased from 0.43 wt% to 0.12 wt%. Lower LODs are directly associated with improved SNRs, enabling the reliable detection of smaller concentrations, a critical factor for trace element analysis in geochemistry [30].

Furthermore, the higher-spectral-resolution instrument enabled the construction of good models (R^2^ ≥ 0.7) for a good number of elements, especially the trace elements Li, Ba, V, Rb, and Sr, whereas not-so-robust models were achieved by the lower-spectral-resolution instrument. In fact, the low-resolution spectrometer was only able to analyze three trace elements (Be, Cr and Pb), and even then, with limitations that need to be further investigated. This result reflects the greater spectral stability and reproducibility of the higher-spectral-resolution instrument, which can minimize pulse-to-pulse fluctuations that weaken the signal–concentration correlation.

The RMSE results achieved with the higher- and lower-spectral-resolution systems are compared in Table 6 with those of a previous study by Ytsma et al. [22], who used PLS on a similar sample set consisting of 2000 rock standards of the Mount Holyoke College (MHC) Mineral Spectroscopy Laboratory using the SciAps Z-300 (higher-resolution) instrument. However, these authors adopted a different protocol, including the collection of three spectra from each sample, each of which was the culmination of a 4 × 3 plasma grid. Despite the difference in the number nature and origin of the samples, the overall performance achieved in this work is surprisingly comparable and appears to capture the range of LIBS accuracies that can be achieved with these instruments for diverse geological samples and diverse modeling approaches. In particular, our work comprises a smaller sample set, incorporates an internal hyperparameter selection scheme (NCV), with LOOCV, and applies the PLS, RF and ANN modeling approaches.

Among the major oxides, there is strong agreement among the RMSE values, which are closely similar. For Li, the results from the higher-spectral-resolution instrument were very similar to those found by Ytsma et al. [22]. For Ba and Cr, the analysis performed in this work shows a superior performance, although the sample set featured substantially lower mean concentrations, which may have influenced the outcomes. In contrast, for Pb, the result was slightly inferior to the one achieved by the lower-spectral-resolution instrument.

Despite the superiority of the higher-spectral-resolution system, the lower-spectral-resolution instrument demonstrated a comparable performance for a few analytes that could be successfully modeled, i.e., the major oxides Al_2_O_3_, MgO, CaO and Na_2_O with relatively high concentrations in the clay matrix (e.g., Al_2_O_3_: 18.6%wt; CaO: 3.53%wt). As the elements present at higher abundances produce sufficiently strong, stable, and prominent spectral lines to correlate with the concentration, the instrumental limitations of the lower-spectral-resolution system (e.g., spectral resolution or SNR) are not critical factors [31].

### 3.4. Synergy Among the Calibration Techniques (PLS, RF, and ANN)

The combined use of PLS, RF, and ANN proved to be a possible and complementary strategy for assessing LIBS data quality. The PLS algorithm consistently showed robustness, providing high-quality models (R^2^ > 0.9) for most of the major-element data acquired by both instruments. The strength of this algorithm lies in its capacity for handling multicollinear spectral data through projection into latent variables, which makes it one of the most stable and interpretable methods [23]. The quantitative performance achieved for major elements deserves further discussion considering the intrinsic plasma-related effects in LIBS, particularly self-absorption (SA). The deviations from linearity observed for major elements can be partially attributed to plasma SA, a well-known phenomenon in LIBS analyses. SA occurs when atoms in the ground state, located in cooler and denser regions of the plasma, partially reabsorb the radiation emitted from hotter regions, which occurs especially for intense spectral lines of elements present at high concentrations. This effect leads to saturation, peak flattening, and line broadening, ultimately resulting in a loss of linearity between the emission intensity and elemental concentration, thereby degrading the quantitative calibration performance. In particular, SA is especially relevant for major elements such as Mg, Al, Ca, and Na, whose concentrations in geological matrices often exceed 1%wt. Consequently, the elevated RMSE values and reduced linear correlations that are achieved for these elements should be interpreted by considering them as intrinsic physical limitations of the LIBS plasma rather than as shortcomings of the modeling strategy. Thus, wavelength intensities rather than integrated peak areas were used in this work. For transitions affected by SA, the line wings often preserve good linearity, whereas the line core is more strongly affected by saturation, as was observed in our previous studies [32].

Differently, both the machine learning algorithms RF and ANN were able to capture complex nonlinear relationships, occasionally outperforming PLS. For example, the ANN achieved an R^2^ of 0.94 for MgO data acquired with the lower-spectral-resolution instrument, which suggests a good predictive power.

The complementarity among the methods used is thus evident; i.e., when one algorithm fails to model an element effectively, the other is often successful. For instance, for TiO_2_ data acquired by the higher-spectral-resolution instrument, all algorithms achieved limited performances (R^2^ ≈ 0.42–0.44), with the RF being more accurate. Yet the consistency across them confirms that Ti transitions possibly suffer from spectral interferences or matrix effects. This multi-algorithm approach adds robustness to the study, allowing for the selection of the best model for each element and enabling cross-validation [33].

One aspect that deserves attention is, however, the limited performance for SiO_2_ data. Although SiO_2_ represented the major constituent of the sample (56.5%wt), it exhibited a limited calibration performance by the higher-spectral-resolution instrument (best R^2^ = 0.72 with PLS) and lower-spectral-resolution instrument (best R^2^ = 0.73 with PLS). As Si features numerous strong spectral lines, a higher R^2^ value is expected; thus, signal saturation of the most intense Si lines might occur, which restricts the analytical sensitivity or matrix effects [34]. Future experiments should address this issue by using a lower laser power.

## 4. Conclusions

In summary, the results of this study confirm the superior performance of the higher-resolution system in terms of sensitivity (lower LODs), versatility (a greater number of calibrated elements), and reliability (higher R^2^ values). Nonetheless, this study also demonstrates that the lower-resolution instrument remains a practically valuable and economically viable tool for the routine quantification of major elements in rocks. Furthermore, the use of multiple calibration algorithms is strongly recommended to account for complex or nonlinear analytical behaviors that may influence LIBS systems.

The implementation of a complementary multi-algorithm approach based on the use of PLS, RFs, and ANNs for the quantitative determination of oxides and major, minor, and trace elements in a set of geochemical reference materials proved to be a powerful and complementary strategy for assessing the LIBS data quality in hLIBS instruments. In particular, PLS consistently represents a robust and interpretable model for linearly correlated data, while the machine learning techniques (RF and ANN) capture complex and nonlinear relationships. This synergy allows for the selection of the most appropriate model for each specific element-instrument combination, enhancing the overall robustness and reliability of the LIBS analytical technique. The results were also compared with those obtained from univariate analysis, demonstrating that the multivariate methods achieved superior performances.

The results of this work demonstrate evidently different performances between the two instruments, primarily attributable to their distinct configurations. The instrument with a higher spectral resolution, broader wavelength range, variable raster grid, and higher pulse energy achieved superior analytical capabilities, enabling the development of calibration models for 16 out of 25 analytes, and exhibited lower average LODs and greater predictive accuracy for both major oxides and trace elements. The other system was effective for the quantitative analysis of 11 analytes out of 25, with a limited performance in trace elements, showing, however, a good performance primarily for major oxides (e.g., Al_2_O_3_, MgO, CaO), the high concentration and strong emission lines of which mitigated its limitations in terms of the spectral resolution and SNR ratio.

In conclusion, the choice between the two types of hLIBS instruments depends heavily on the specific application requirements. For applications demanding high sensitivity, broader elemental coverage, and higher quantification accuracy for trace elements, the higher-spectral-resolution instrument shows a superior performance; however, the lower-spectral-resolution instrument remains a viable and cost-effective option for the rapid field screening and quantification of major elements.

Finally, the results of this work underscore the maturity of hLIBS as a powerful tool for in situ geochemical analysis and highlight the critical importance of instrumental specifications and robust data processing in achieving optimal analytical results. Furthermore, in the future, the algorithm and hyperparameter selection used in this work will be trained by an automated (AI-driven) system and will hopefully be incorporated directly into the software of the handheld instruments to allow for the achievement of a direct in situ response, making their use easier even for non-trained people.

## Figures and Tables

**Table 1:** Overview of the main characteristics, as reported by the manufacturers, of the two hLIBS instruments used in this study and some of their physical and operational parameters.

Brand	Laser Type	Laser Class	Laser Repetition Rate	Laser Energy Per Pulse	Wavelength Range (nm)	Spectral Resolution FWHM * (nm)
B&W Tek NanoLIBS	Nd:YAG 1064 nm	3B	5 kHz	150 µJ	180–800	0.38–0.5
SciAps *Z-300*	Nd:YAG 1064 nm	3B	50 Hz	5–6 mJ	190–950	0.18–0.35
**Brand**	**Detector Type**	**Laser Crater** **Diameter (μm)**	**Analysis** **Atmosphere**	**Dimensions** **L × H × W (cm)**	**Weight with** **Battery (kg)**	**Battery Type** **Operating Time**
B&W Tek NanoLIBS	CCD	300	Ambient	26.5 × 30.4 × 10	1.8	Li-ion >4 h
SciAps *Z-300*	CCD	100	Ambient or argon purge	21 × 29.2 × 11.4	1.8	Li-ion 4–7 h

* The exact value depends on the spectral region and application, so these numbers are approximate.

**Table 2:** Data related to the processing and calculation of the LOD for the higher-spectral-resolution hLIBS instrument. The LOD and Std background have the same units as the corresponding compounds (wt% for oxides and mg/kg for elements), whereas R^2^ is dimensionless.

	Analyte	Conc. Average	Conc. Range	LOD	R^2^	Std Background	Slope	Emission Line (nm)
Oxides (wt%)	**SiO_2_**	56.5	36.45–75.80	3.19	0.69	0.39	0.37	390.53
	**Al_2_O_3_**	18.6	2.9–59.2	0.83	0.94	0.39	1.41	394.53
	**MgO**	4.87	0.01–35.21	0.94	0.98	0.39	1.25	517.50
	**CaO**	3.53	0.04–15.9	1.01	0.97	0.39	1.15	865.43
	**Na_2_O**	2.44	0.04–15.9	0.53	0.97	0.39	2.21	820.00
	**K_2_O**	3.34	0.02–12.81	0.35	0.98	0.39	3.31	693.63
	**TiO_2_**	0.77	0.01–2.61	0.12	0.72	0.39	9.40	375.33
	**Fe_2_O_3_**	5.95	0.075–19.6	1.57	0.88	0.39	0.74	254.17
Elements (mg/kg)	**Li**	40.79	1–110	1.27	0.84	0.43	1.01	670.43
	**Ba**	117.1	6–385	22.74	0.69	0.43	0.06	454.83
	**V**	91.3	0.2–340	33.34	0.85	0.41	0.04	322.20
	**Cr**	44.72	2–140	1.94	0.77	0.34	0.52	279.30
	**Rb**	76.07	1–238	16.05	0.99	0.48	0.089	779.63
	**Sr**	147.69	3–570	51.96	0.93	0.40	0.023	420.93

**Table 3:** Data related to the processing and calculation of the LOD for the lower-spectral-resolution hLIBS instrument. The LOD and Std background have the same units as the corresponding compounds (wt% for oxides and mg/kg for elements), whereas R^2^ is dimensionless.

	Analyte	Conc. Average	Conc. Range	LOD	R^2^	Std Background	Slope	Emission Line (nm)
Oxides (wt%)	**SiO_2_**	56.5	36.45–75.80	1.62	0.80	0.13	0.24	250.00
	**Al_2_O_3_**	18.6	2.9–59.2	1.57	0.95	0.13	0.25	394.77
	**MgO**	4.87	0.01–35.21	1.76	0.92	0.13	0.22	293.91
	**CaO**	3.53	0.04–15.9	0.52	0.92	0.13	0.76	392.79
	**Na_2_O**	2.44	0.04–15.9	0.23	0.94	0.13	1.69	590.08
	**K_2_O**	3.34	0.02–12.81	2.21	0.84	0.13	0.18	767.07
	**TiO_2_**	0.77	0.01–2.61	0.43	0.66	0.13	0.92	375.70
	**Fe_2_O_3_**	5.95	0.075–19.6	1.60	0.77	0.13	0.25	275.78
Elements (mg/kg)	**Be**	5.09	0.2–35	1.90	0.67	0.14	0.22	249.87
	**Cr**	44.72	2–140	3.04	0.73	0.10	0.10	277.76
	**Pb**	39.48	2–240	14.54	0.55	0.143	0.029	287.41

**Table 4:** The LOD, R^2^ and RMSE values achieved by the application of the univariate analysis, PLS, RF and ANN algorithms to data acquired by the higher-spectral-resolution hLIBS instrument. The best-performing metrics from the validation are indicated in bold. The LOD and RMSE have the same units as the corresponding compounds (wt% for oxides and mg/kg for elements), whereas the R^2^ is dimensionless.

				Univariate		PLS		RF		ANN	
	Analyte	Conc. Range	LOD	R^2^	RMSE	R^2^	RMSE	R^2^	RMSE	R^2^	RMSE
Oxides (wt%)	**SiO_2_**	36.45–75.80	3.19	0.36	10.22	**0.70**	**7.52**	0.4	10.28	0.43	9.69
	**Al_2_O_3_**	2.9–59.2	0.83	0.79	5	**0.93**	**2.92**	0.75	7.26	0.83	5.30
	**MgO**	0.01–35.21	0.94	0.93	2.66	**0.95**	**2.22**	0.88	4.32	0.93	2.90
	**CaO**	0.04–15.9	1.01	0.91	1.66	0.92	1.78	**0.94**	**1.62**	0.93	1.71
	**Na_2_O**	0.04–15.9	0.53	0.87	0.88	**0.89**	**0.86**	0.80	1.41	0.88	1.02
	**K_2_O**	0.02–12.81	0.35	**0.96**	**0.71**	**0.92**	**0.99**	0.85	1.40	0.89	1.21
	**TiO_2_**	0.01–2.61	0.12	0.01	0.72	0.42	0.58	0.44	**0.55**	0.40	0.71
	**Fe_2_O_3_**	0.075–19.6	1.57	0.62	3.08	**0.73**	**2.67**	0.71	3.02	0.70	3.10
Elements (mg/kg)	**Li**	1–110	1.25	0.59	21.46	**0.80**	**15.41**	0.58	22.54	0.75	17.46
	**Ba**	6–385	22.74	0.36	143.57	0.63	71.43	0.73	64.17	**0.76**	**62.36**
	**V**	0.2–340	33.34	0.54	61.75	**0.84**	**36.77**	0.79	49.40	0.81	47.10
	**Cr**	2–140	1.94	0.07	38.4	**0.69**	**23.51**	0.67	24.16	0.68	25.31
	**Rb**	1–238	16.05	0.92	20.88	**0.94**	**18.8**	0.92	24.55	0.93	23.85
	**Sr**	3–570	51.96	0.75	91.63	0.85	68.93	0.83	80.2	**0.92**	**47.20**

**Table 5:** The LOD, R^2^ and RMSE values achieved by application of the univariate analysis, PLS, RF and ANN algorithms to data acquired by the lower-spectral-resolution instrument. The best-performing metrics from the validation are indicated in bold. The LOD and RMSE have the same units as the corresponding compounds (wt% for oxides and mg/kg for elements), whereas R^2^ is dimensionless.

				Univariate		PLS		RF		ANN	
	Analyte	Conc. Range	LOD	R^2^	RMSE	R^2^	RMSE	R^2^	RMSE	R^2^	RMSE
Oxides (wt%)	**SiO_2_**	36.45–75.80	1.62	0.58	8.29	**0.73**	**7**	0.65	7.58	0.58	8.40
	**Al_2_O_3_**	2.9–59.2	1.57	0.89	3.67	**0.94**	**2.9**	0.85	6.88	0.77	5.39
	**MgO**	0.01–35.21	1.76	0.82	4.25	0.72	4.48	0.82	4.23	**0.94**	**2.25**
	**CaO**	0.04–15.9	0.52	0.74	2.69	0.75	2.95	0.93	1.43	**0.94**	**1.34**
	**Na_2_O**	0.04–15.9	0.23	0.82	1.08	**0.93**	**0.72**	0.79	1.41	0.89	0.93
	**K_2_O**	0.02–12.81	2.21	0.20	3.28	**0.54**	**2.46**	0.48	2.76	0.44	3.02
	**TiO_2_**	0.01–2.61	0.43	0.22	0.67	0.40	0.75	0.43	0.68	**0.44**	**0.54**
	**Fe_2_O_3_**	0.075–19.6	1.60	0.29	4.35	0.29	5.44	**0.38**	**4.11**	0.34	4.70
Elements (mg/kg)	**Be**	0.2–35	1.90	0.08	10.32	**0.94**	**5.21**	0.70	8.62	0.67	8.79
	**Cr**	2–140	3.04	**0.63**	**21.51**	**0.48**	**31.12**	0.45	35.2	0.43	39.7
	**Pb**	2–240	14.54	0.30	69.72	0.25	84.9	0.36	75.3	**0.81**	**44.21**

**Table 6:** A comparison of the validation errors (RMSE) achieved with the higher-spectral-resolution and lower-spectral-resolution instruments and the literature data from Ytsma et al. [22].

Oxides	Higher Spectral Resolution (wt%)	Lower Spectral Resolution (wt%)	Ytsma et al. [22] (wt%)
SiO_2_	7.52	7.00	8.61
Al_2_O_3_	2.95	2.90	3.38
MgO	2.22	2.25	4.41
CaO	1.62	1.34	3.08
Na_2_O	0.86	0.72	1.09
K_2_O	0.99	2.46	1.52
TiO_2_	0.55	0.54	0.77
Fe_2_O_3_	2.67	4.11	3.84
**Elements**	**(mg/kg)**	**(mg/kg)**	**(mg/kg)**
Li	15.41	N/A	21
Be	N/A	5.21	N/A
Ba	62.36	N/A	565
V	36.77	N/A	N/A
Cr	23.51	31.12	239
Rb	18.80	N/A	N/A
Sr	47.20	N/A	N/A
Pb	N/A	44.21	19

N/A: No prediction (<LOD).

## Data Availability

All data analyzed during this study are included in this published article for any further request you’re welcome to get in touch.

## Supplementary Materials

The following supporting information can be downloaded at: https://www.mdpi.com/article/10.3390/s26031076/s1, Figure S1: Calibration curves–Z300 LIBS–oxides; Figure S2: Calibration curves–Z300 LIBS–elements; Figure S3: Calibration curves–NanoLIBS–oxides; Figure S4: Calibration curves–NanoLIBS–elements; Figure S5: Validation curves–Z300 LIBS–oxides; Figure S6: Validation curves–Z300 LIBS–elements; Figure S7: Validation curves–NanoLIBS–oxides; Figure S8: Validation curves–NanoLIBS–elements; Figure S9: Evolution of the root-mean-square error (RMSE) for calibration and validation datasets as a function of the number of training epochs for MgO measured in the lower-spectral-resolution instrument. The convergence of both curves, together with the analysis of the LOOCV results (R^2^ = 0.94), indicates a low likelihood of overfitting and the high generalization capability of the model for predicting external samples; Figure S10: Evolution of the root-mean-square error (RMSE) for calibration and validation datasets as a function of the number of training epochs for CaO measured in the lower-spectral-resolution instrument. The convergence of both curves, together with the analysis of the LOOCV results (R^2^ = 0.94), indicates a low likelihood of overfitting and the high generalization capability of the model for predicting external samples. Table S1: List of the 21 Certified Reference Materials and their origin; Table S2: Oxides and elements chemical composition for the 21 Certified Reference Materials; Table S3: Optimal hyperparameter values selected for each classifier (PLS, RF, and ANN) during the LOOCV procedure.
