# Supplementary material for: Machine Learning-Enhanced Evaluation of Handheld Laser-Induced Breakdown Spectroscopy (LIBS) Analytical Performance for Multi-Element Analysis of Rock Samples
Source: Sensors (Basel). 2026 Feb 6;26(3):1076. doi: 10.3390/s26031076 (PMC12900111; doi:10.3390/s26031076)
Supplement: Supplementary file 1 [file sensors-26-01076-s001.zip › Figs S1-S2-S3-S4-S5-S6-S7-S8 Univariate Calibration and Validation Curves.pdf]

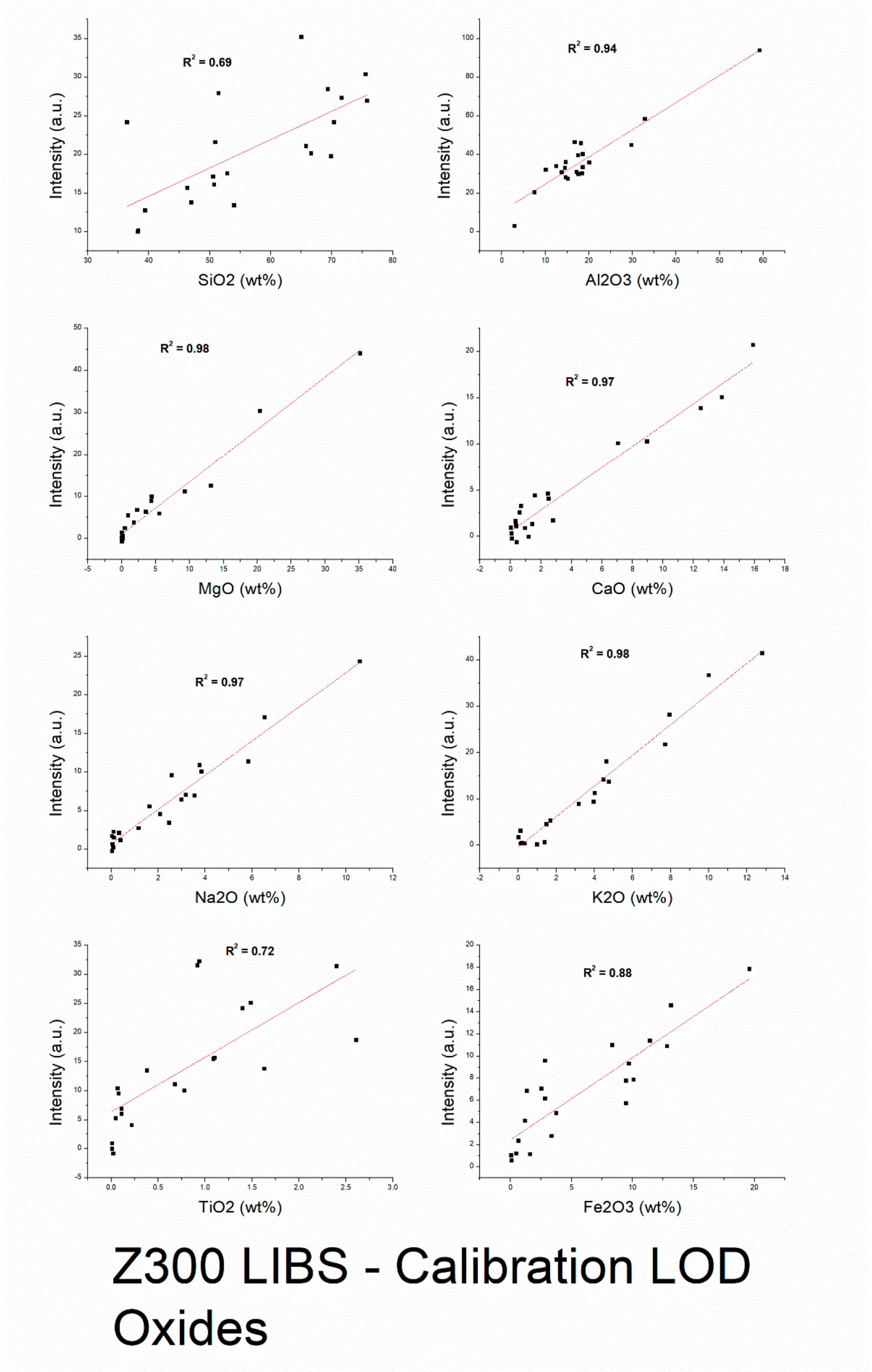

Figure S1: Calibration Curves - Z300 LIBS – Oxides

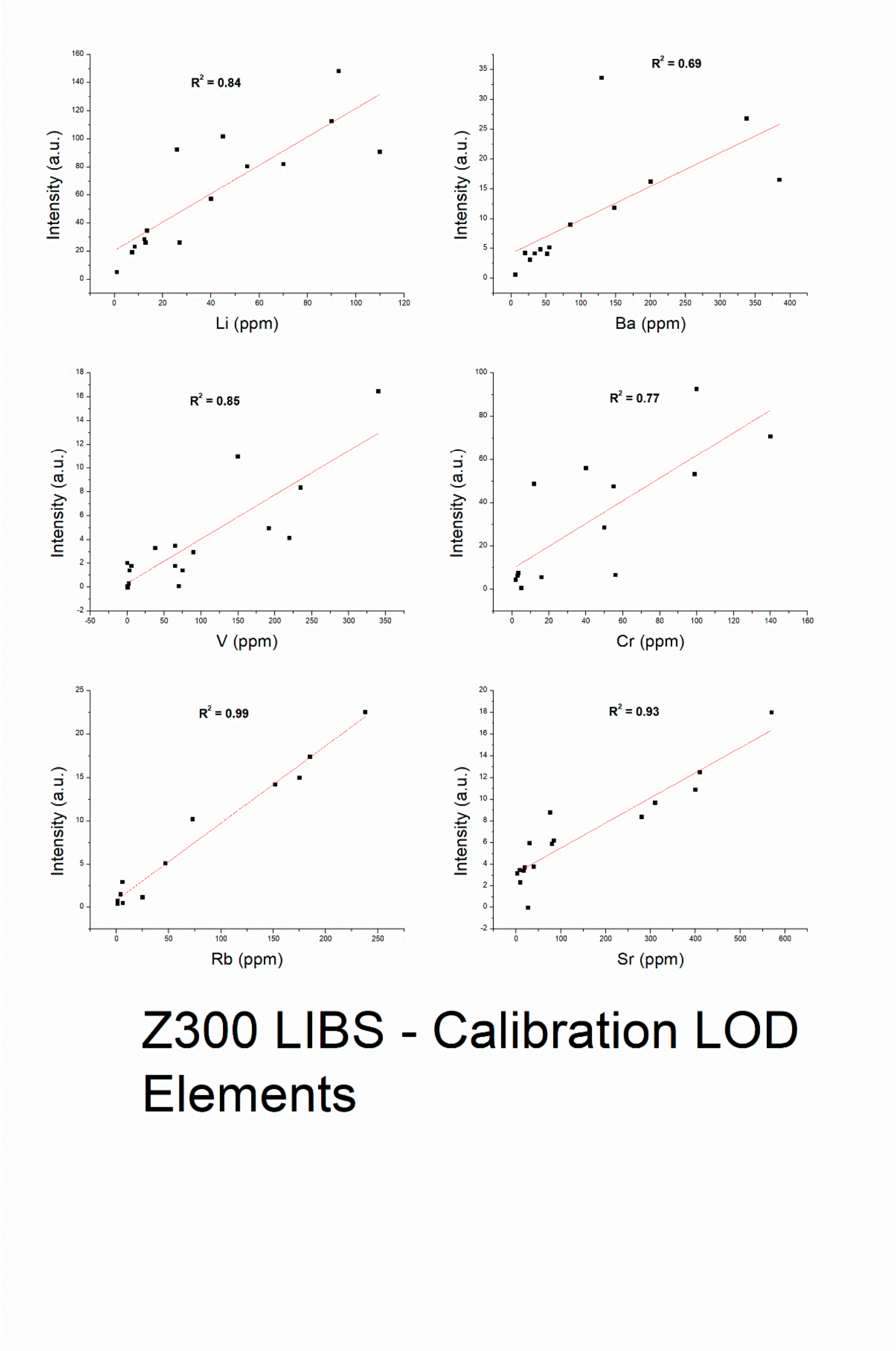

Figure S2: Calibration Curves – Z300 LIBS – Elements

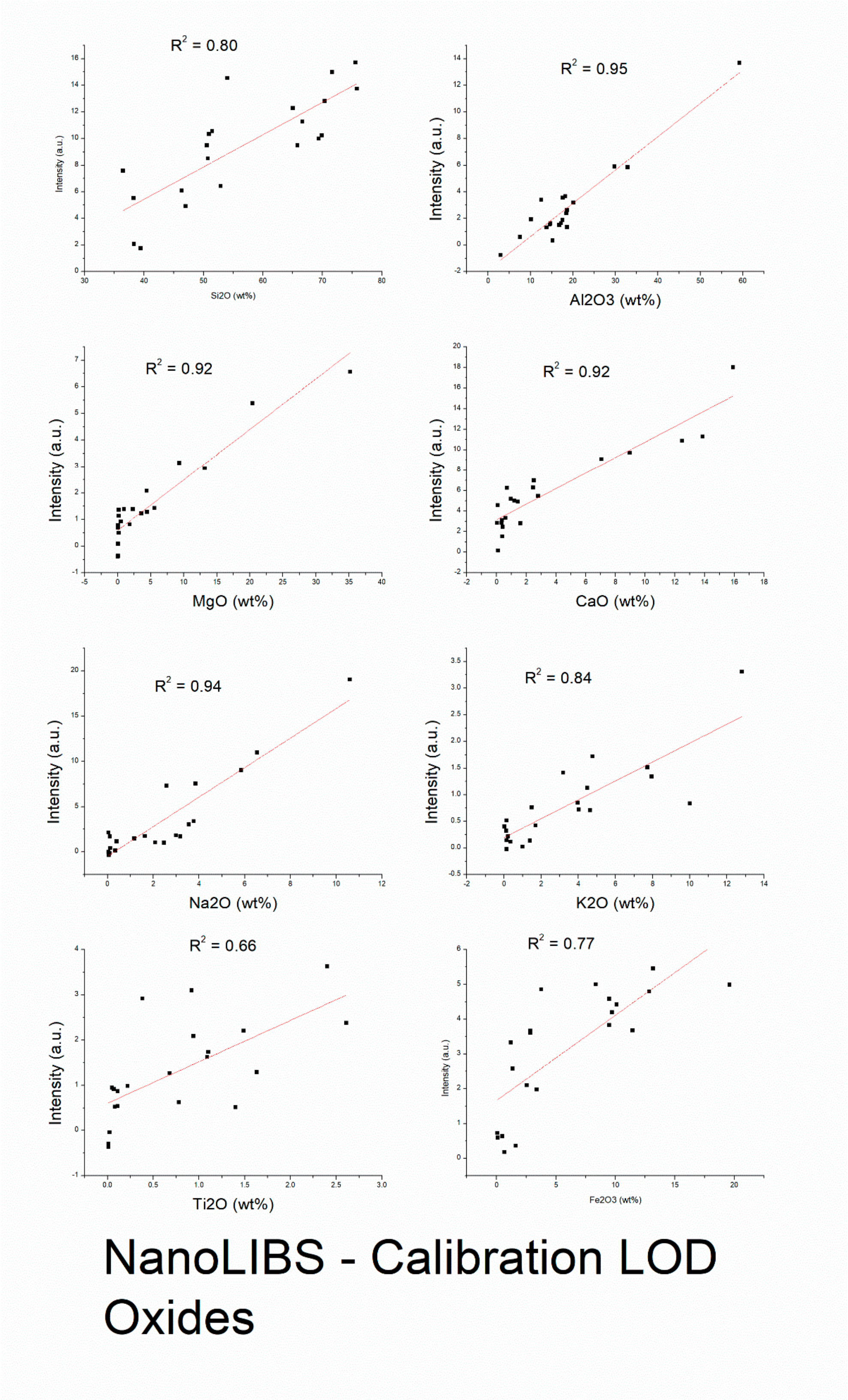

Figure S1: Calibration Curves – NanoLIBS – Oxides

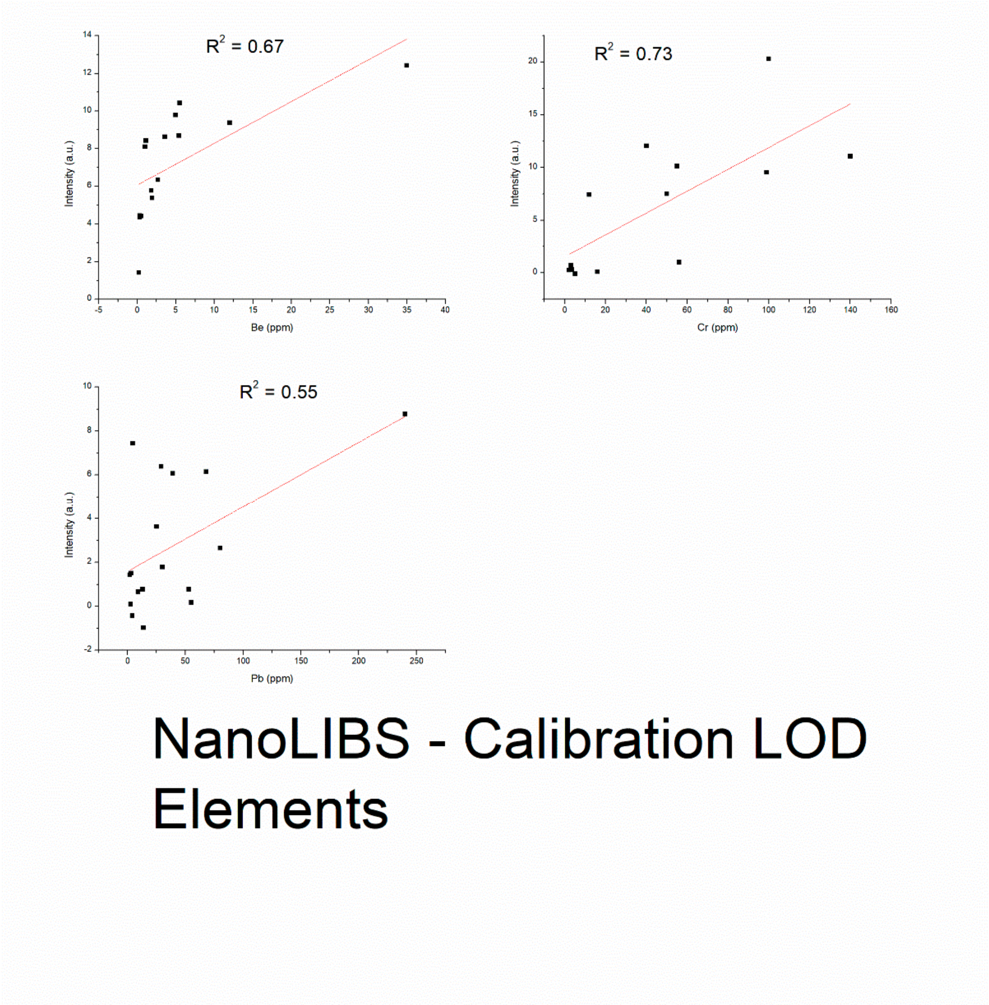

Figure S2: Calibration Curves – NanoLIBS - Elements

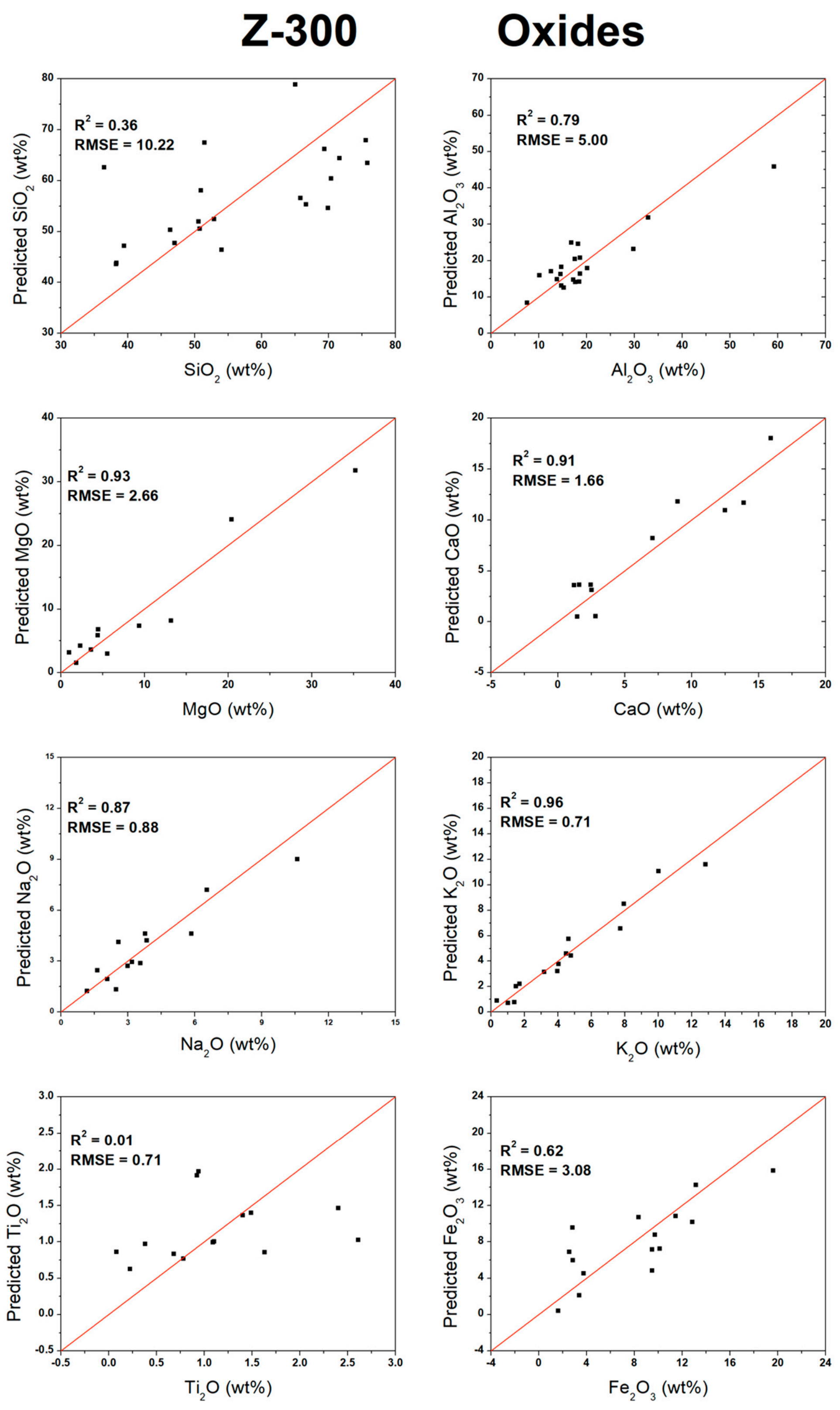

Figure S3: Validation Curves – Z300 LIBS – Oxides

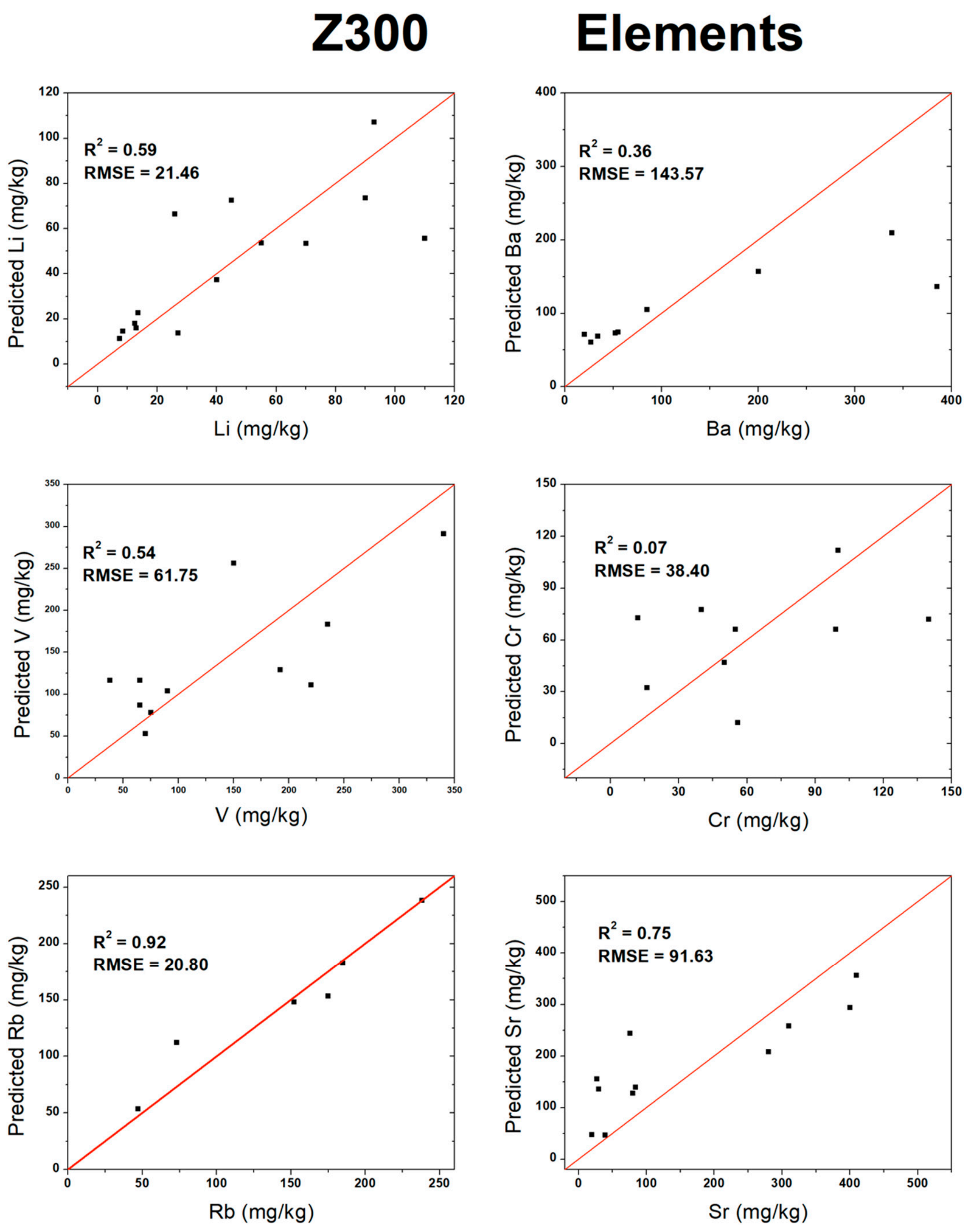

Figure S4: Validation Curves – Z300 LIBS – Elements

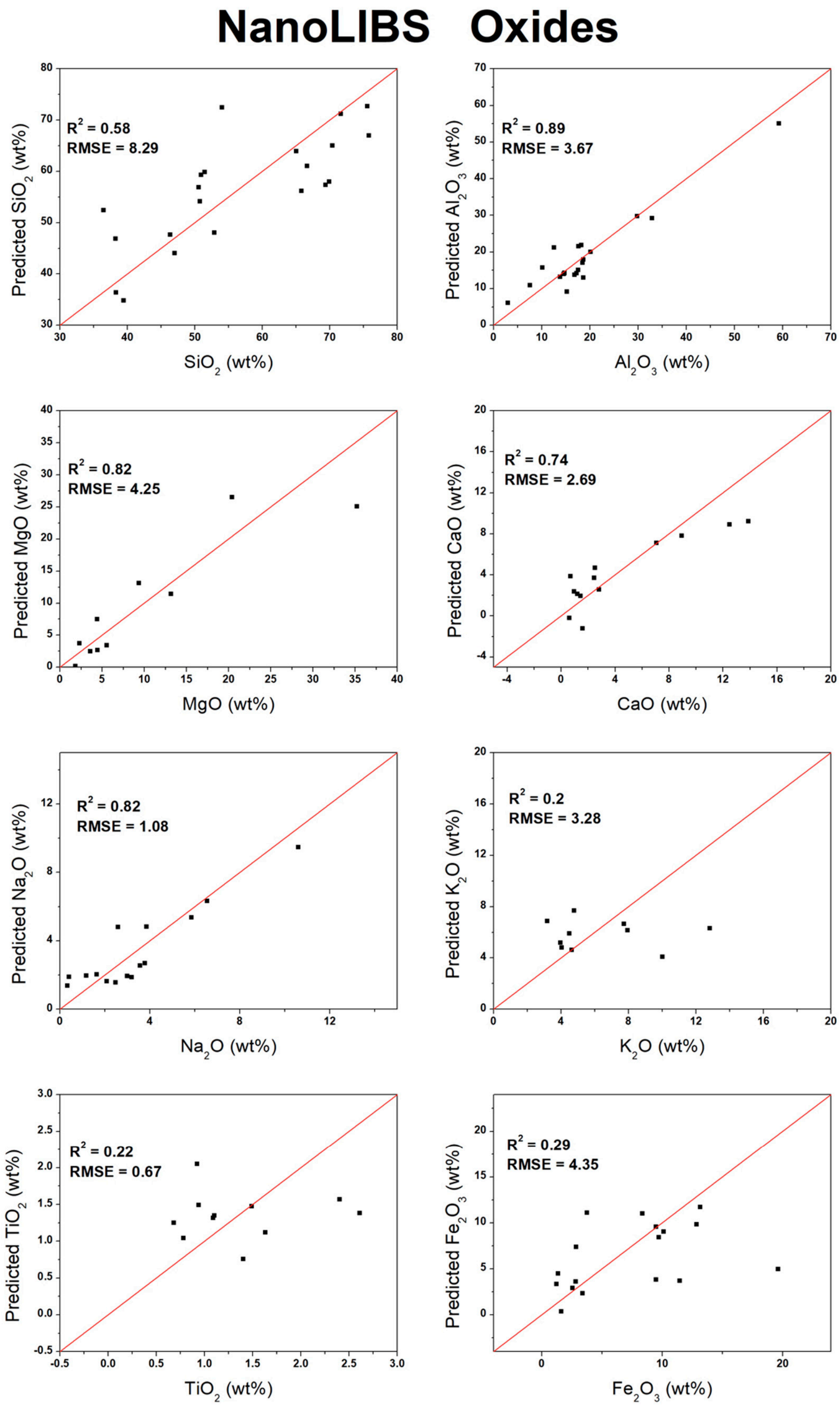

Figure 5S: Validation Curves – NanoLIBS – Oxides

NanoLIBS   Elements

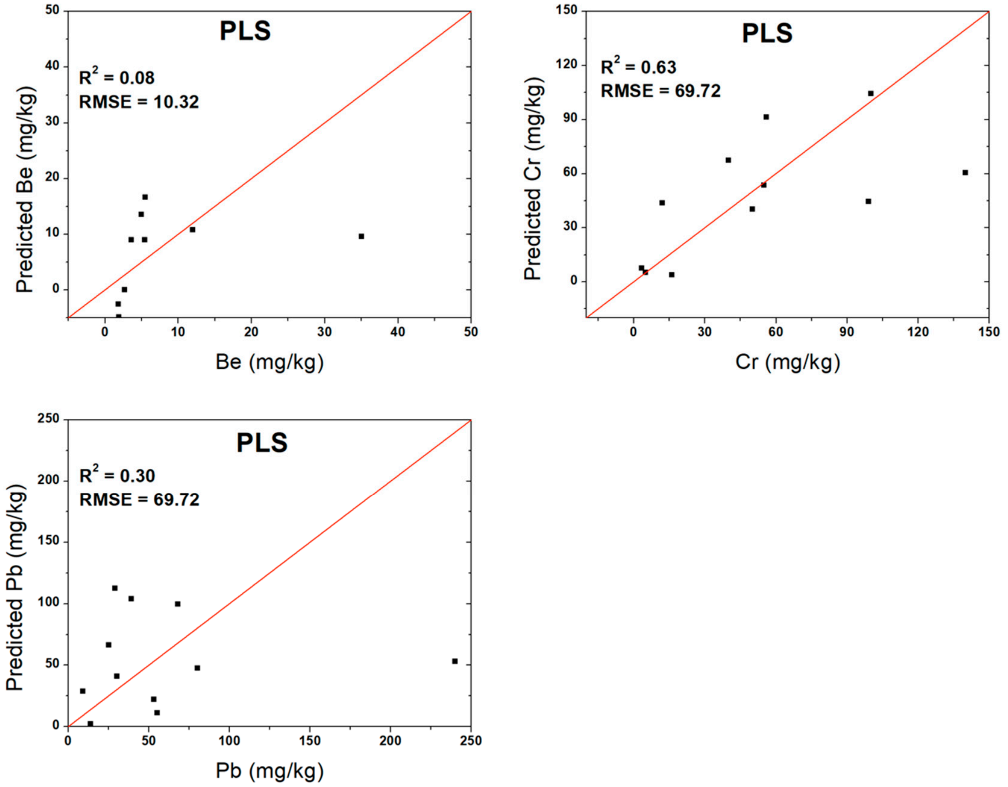

Figure S6: Validation Curves – NanoLIBS - Elements
