# Supplementary material for: Machine Learning-Enhanced Evaluation of Handheld Laser-Induced Breakdown Spectroscopy (LIBS) Analytical Performance for Multi-Element Analysis of Rock Samples
Source: Sensors (Basel). 2026 Feb 6;26(3):1076. doi: 10.3390/s26031076 (PMC12900111; doi:10.3390/s26031076)
Supplement: Supplementary file 1 [file sensors-26-01076-s001.zip › Figs S9-S10 Error Graphs.pdf]

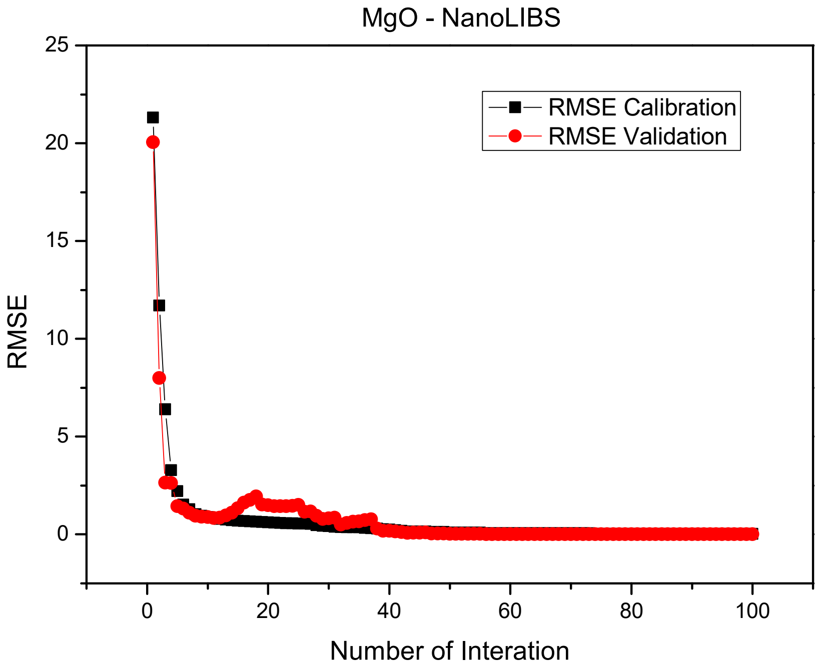

Figure S9: Evolution of the root mean square error (RMSE) for calibration and validation datasets as a function of the number of training epochs for MgO measured in the lower spectral resolution instrument. The convergence of both curves, together with the analysis of the LOOCV results (R^2 = 0.94), indicates a low likelihood of overfitting and a high generalization capability of the model for predicting external samples.

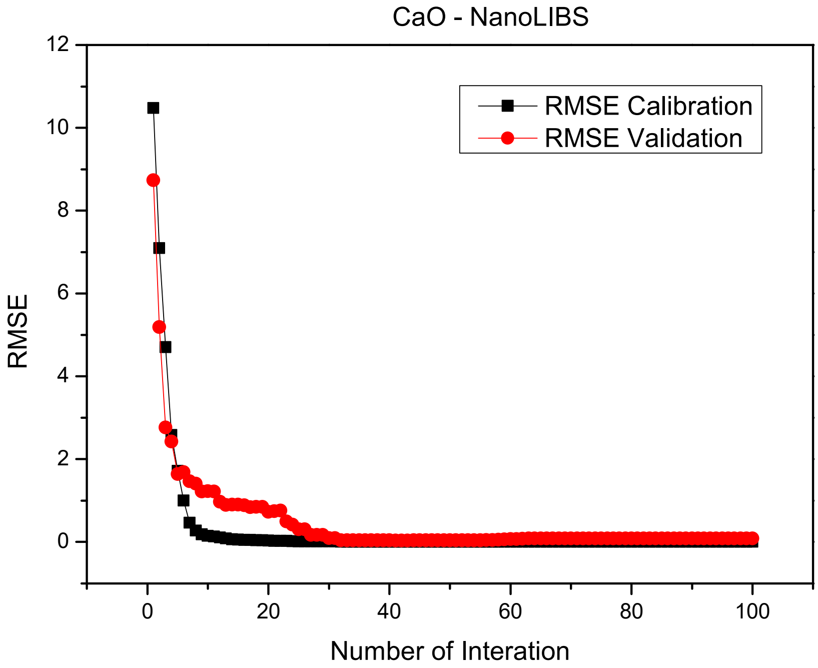

Figure S10: Evolution of the root mean square error (RMSE) for calibration and validation datasets as a function of the number of training epochs for CaO measured in the lower spectral resolution instrument. The convergence of both curves, together with the analysis of the LOOCV results (R^2 = 0.94), indicates a low likelihood of overfitting and a high generalization capability of the model for predicting external samples.
