# Supplementary material for: Machine Learning-Enhanced Evaluation of Handheld Laser-Induced Breakdown Spectroscopy (LIBS) Analytical Performance for Multi-Element Analysis of Rock Samples
Source: Sensors (Basel). 2026 Feb 6;26(3):1076. doi: 10.3390/s26031076 (PMC12900111; doi:10.3390/s26031076)
Supplement: Supplementary file 1 [file sensors-26-01076-s001.zip › Table S1.pdf]

Table S1

Certified Reference Material		Origin
1- AC-E	Granite	Ailsa Craig Island, Scotland (UK)
2- AL-I	Albite	Pinzolo (Trento), Italy
3- AN-G	Anorthosite	West Greenland (Denmark)
4- ARG-CNR	Kaolinitic Clay	Italy
5- BAR1	Kaolinitic Clay	Italy
6- BAR2	Kaolinitic Clay	Italy
7- BE-N	Basalt	Meurthe-et-Moselle (France)
8- DR-N	Diorite	Champ de feu, Vosges (France)
9- DT-N	Disthene	Nanga, Eboko (Cameroun)
10- FK-N	Potash Feldspar	Madras, Tamil Nadu (India)
11- GA	Granite	Andlau, Bas-Rhin (France)
12- GH	Granite	Massif Taourit (Hoggar, Algerie)
13- GL-O	Glauconite	Cauville-sur-Mer, Normandie (France)
14- GS-N	Granite	Senones, Vosges (France)
15- MA-N	Granite	Beauvoir, Massif Central (France)
16- Mica-MG	Phlogopite	Bekily (Southern Madagascar)
17- PM-S	Microgabbro	Ailsa Craig Island, Scotland (UK)
18- SYF	Clay	Italy
19- UB-N	Serpentine	Col des Bagenelles, Vosges (France)
20- WS-E	Dolerite	Ailsa Craig Island, Scotland (UK)
21- ZW-C	Zinnwaldite	Zinnwald (Czech Republic)
